# Pattern of Vascular Anomalies Associated With Sinus Venosus Atrial Septal Defect

**DOI:** 10.7759/cureus.21892

**Published:** 2022-02-04

**Authors:** Ali Akbar, Ijaz Hussain, Haseen Dil Wazir, Yasir Rehman, Saadia Ilyas, Sohail Khan, Tauseef Ahmed, Abdul Moeed Khan, Ikram Ullah, Aamir Afridi

**Affiliations:** 1 Pediatric Cardiology, Lady Reading Hospital MTI (Medical Teaching Institute), Peshawar, PAK; 2 Pediatric Cardiology, Peshawar Institute of Cardiology, Peshawar, PAK

**Keywords:** assessment, children, vascular anomalies, sinus venosus atrial septal defect, ct angiography

## Abstract

Objective

To evaluate children with sinus venosus atrial septal defect (SV-ASD) for associated vascular anomalies.

Methodology

A total of 72 children with sinus venosus atrial septal defect with partial anomalous pulmonary venous return who presented to pediatric cardiology unit, Lady Reading Hospital Peshawar, from January 2019 till June 2021 were included in this cross-sectional study. Diagnosis of sinus venosus atrial septal defect was confirmed through two-dimensional (2D) and Doppler echocardiography performed by a pediatric cardiologist. Cardiac CT angiography was performed and assessed by a pediatric cardiac interventionist and radiologist. Patients were managed according to standard protocols and guidelines. The data were entered and analyzed with Statistical Package for the Social Sciences (SPSS) version 20. Percentages were used to express frequencies.

Results

Mean age was 8.3 ± 2.7 years (interquartile range (IQR): two months to 18 years). There were 37 (51.4%) male and 35 (48.6%) female patients. Out of 72 patients, 64 (88.8%) patients had superior sinus venosus atrial septal defect, while inferior sinus venosus atrial septal defect was found in eight (11.1%) patients. In six (8.3%) patients, associated secundum atrial septal defect was identified. Bilateral superior vena cava was found in seven (9.7%) patients. Left aortic arch was seen in 70 (97.2%) patients, whereas two (2.7%) patients had right aortic arch.

Conclusion

Sinus venosus ASD is a rare type of atrial septal defect which is also associated with both pulmonary and systemic vascular anomalies. Diagnosing these vascular anomalies is of paramount importance before any corrective intervention can be done. Recognizing the pattern of these anomalies should be known to every interventional cardiologist, radiologist, and cardiac surgeon. Echocardiography alone is not a good tool to assess these extracardiac structures. Imaging modalities like CT angiography and MRI have refined our preoperative workup which is essential for the ultimate outcome of the corrective intervention.

## Introduction

Atrial septal defect (ASD) accounts for 8% to 10% of all congenital heart diseases with an incidence of 56 per 100,000 live births [1]. Sinus venosus ASD (SV-ASD) accounts for 5% of all ASDs [2]. Male to female ratio of SV-ASD is reported 1:1 [3]. Mostly, ASD is acquired as a sporadic condition, but familial modes of inheritance have also been observed. Risk of congenital heart disease in offspring of a mother with ASD is around 8% to 10% [4]. ASD may result due to mutation of regulatory gene or their target sarcomere receptor gene, mutation in transcription factors, or associated with syndromes like Noonan, Down, Williams, Kabuki, and Klinefelter syndrome. In addition, maternal diseases and exposure to environmental risk factors like maternal diabetes, phenylketonuria, drugs like nonsteroidal anti-inflammatory drugs (NSAIDs), anticonvulsants, smoking, and alcohol are associated with increased risk of ASD in offspring [5-8].

Most patients with ASD are asymptomatic and may remain undiagnosed until later in life. They may come to medical attention due to abnormal auscultatory findings or diagnostic studies such as ECG, chest radiograph, or echocardiogram. Very rarely, some infants with ASD may present with features of pulmonary overcirculation, recurrent respiratory infections, and failure to thrive [9]. Partial anomalous pulmonary venous return (PAPVR) is present in almost 90% of patients with SV-ASDs; patients with total anomalous pulmonary venous return (TAPVR) usually present early with cyanosis [10].

Diagnosis of SV-ASD can be done by echocardiography, but assessment and mapping of associated vascular anomalies may be difficult requiring cardiac catheterization. Cardiac catheterization is an invasive procedure and may be associated with complications associated with anesthesia, bleeding from catheter site, and hematoma formation. Computerized tomography (CT) and magnetic resonance imaging are being used more recently in diagnosing and evaluating congenital heart diseases [11,12]. Multislice CT has a high spatial and temporal resolution and multiplanar reconstruction capabilities to characterize ASDs and pulmonary venous anomalies [13]. Surgical correction of SV-ASD with anomalous pulmonary return involves closure of ASD and redirection of anomalous pulmonary veins into the left atrium [14]. Early experience in selected patients using a two-stage simulation strategy, percutaneous correction of SV-ASD with PAPVR, has been found to be feasible and safe short-term outcome [15].

Detailed assessment of vascular anatomy affects the outcome of either surgical or percutaneous approach for correction of SV-ASD with associated PAPVR. This study is meant to recognize different patterns of vascular anomalies associated with SV-ASD.

## Materials and methods

Study design

This descriptive cross-sectional study was conducted at Lady Reading Hospital, Peshawar, Pakistan. Data collection was started in January 2019 and completed in June 2021.

Inclusion and exclusion criteria

All patients irrespective of age and gender with sinus venosus ASD and suspected pulmonary venous anomalies on echocardiography were included in study. Patients with complex congenital heart defects were excluded from this study. 

Data collection

All the cases of SV-ASD with anomalous pulmonary venous return who presented to outdoor and indoor department of Pediatric Cardiology were included in the study. Diagnosis of SV-ASD and anomalous pulmonary venous return was made on echocardiography performed by a pediatric cardiologist, and CT angiography was done for confirmation and evaluation of vascular anomalies. Detailed history and examination was performed. SV-ASDs were classified as superior and inferior sinus venosus ASD. Additionally, associated secundum ASD, persistent left superior vena cava, and position of arch of aorta were also documented. Gender and age of the patient were also documented.

Statistical analysis 

All numerical values obtained from each item of the data collection sheet as well as the demographic data were computed and presented by simple descriptive statistical tests, frequency, and percentage. The IBM Statistical Package for the Social Sciences (SPSS) Statistics for Windows, Version 21.0, (Released 2012, IBM Corp, Armonk, New York) was used for data analysis.

Ethical considerations

This study was approved by the Institutional Review Board of Lady Reading Hospital, Peshawar, Pakistan with the approval number 225/LRH/MTI.

## Results

**Figure 1:**
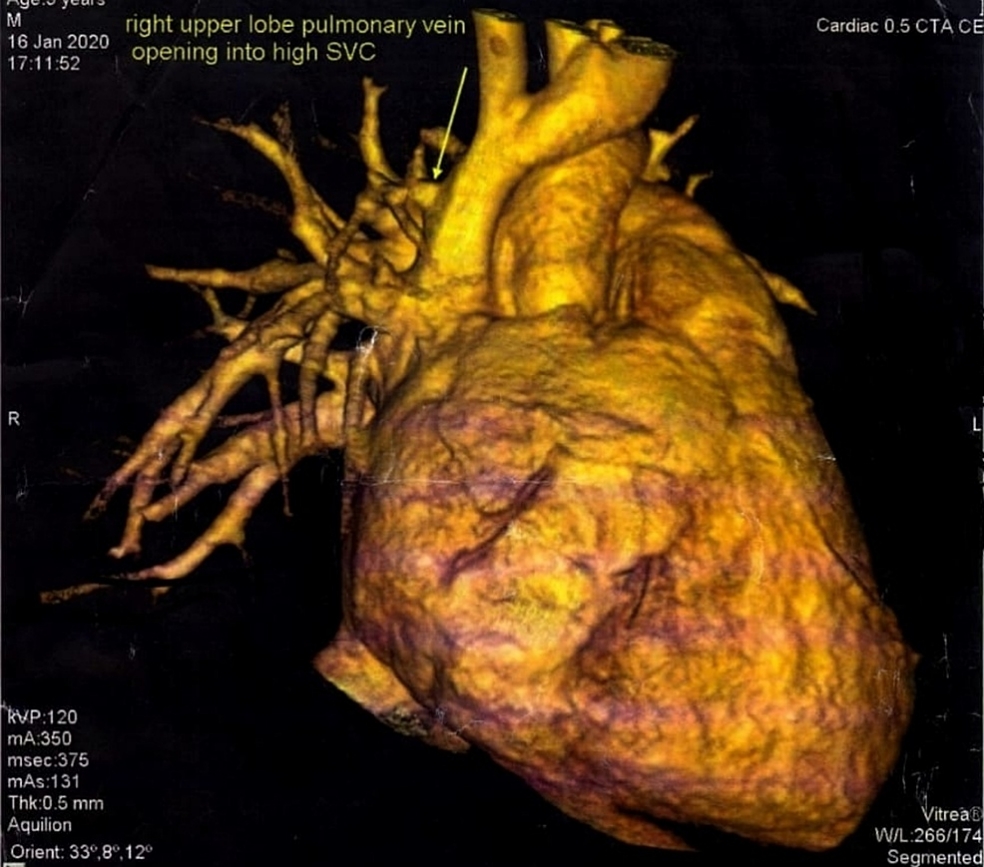
Anomalous right upper pulmonary vein opening into high right superior vena cava M: male patient, SVC: superior vena cava, CTA: computed tomography angiography, CE: contrast enhanced, R: right side, L: left side, W/L: window level.

From January 2019 to June 2021, a total of 72 patients with sinus venosus ASD and anomalous pulmonary venous return were enrolled in the study through outdoor and indoor department. Out of which, 37 (51.3%) patients were male and 35 (48.6%) patients were female (Table 1). Age range of cases was from two months to 14 years. Mean age was 2.8 ± 2.7 years. Superior SV-ASD was the most common type found in 64 (88.8%) patients, while inferior SV-ASD was found in eight (11.1%) patients (Table 2). In seven (9.7%) patients, anomalous vein was draining into upper and middle part of superior vena cava (SVC) (Figure 1). Anomalous vein draining into inferior vena cava (IVC) was seen in nine (12.5%) patients. In one (1.3%) case, anomalous vein was seen opening into right atrium. In 55 (76.3%) patients, anomalous pulmonary vein was seen draining at SVC and right atrial junction (Table 3). In six (8.3%) patients, associated secundum atrial septal defect was identified. Bilateral superior vena cava was found in seven (9.7%) patients (Table 4). Left aortic arch was seen in 70 (97.2%) patients, while two (2.7%) patients had right aortic arch (Table 5).

**Table 1:** Gender wise distribution

Gender	N (%)
Male	37 (51.3%)
Female	35 (48.6%)
Total	72 (100%)

**Table 2:** Types of sinus venosus atrial septal defect SV-ASD: sinus venosus atrial septal defect.

Type of SV-ASD	N (%)
Superior SV-ASD	64 (88.8%)
Inferior SV-ASD	8 (11.1%)
Total	72 (100%)

**Table 3:** Drainage site of anomalous pulmonary vein SVC: superior vena cava, RA: right atrium, IVC: inferior vena cava.

Anomalous vein drainage	N (%)
SVC RA junction	55 (76%)
IVC	9 (12.5%)
SVC (upper and middle part)	7 (9.7%)
Right atrium	1 (1.3%)

**Table 4:** SVC anatomy SVC: superior vena cava.

Superior vena cava	N (%)
Bilateral SVC	7 (9.7%)
Right SVC	65 (90.2%)

**Table 5:** Aortic arch sidedness

Aortic arch	N (%)
Left aortic arch	70 (97.4%)
Right aortic arch	2 (2.7%)
Total	72 (100%)

## Discussion

Although SV-ASD is a simple congenital heart defect, vascular anomalies in the form of PAPVR and persistent left SVC in addition to right SVC are commonly observed in patients with SV-ASD [16]. Therefore, repair of SV-ASD should be performed carefully to avoid obstruction to the flow of SVC or PAPVR. Selection of surgical approach depends upon the anatomy of anomalous pulmonary venous return, and a number of reconstructive options may be adopted for correction of these defects [17]. SV-ASD can be diagnosed using echocardiography, but defining the course of anomalous vein may be difficult or sometimes not possible due to poor acoustic window [11].

For evaluation of congenital heart diseases, echo and cardiac catheter angiography are considered as primary diagnostic tools, whereas CT has got a complementary role in cardiac imaging. Echocardiography besides being operator dependent may not be sufficient for evaluation of extracardiac vascular anatomy due to limited acoustic window. Cardiac catheter angiography is an invasive procedure and is limited by its two-dimensional (2D) nature along with difficulties in evaluation of systemic and pulmonary vascular system simultaneously [18]. CT angiography is a reliable tool for assessment of detailed anatomy of anomalous vessels, but knowledge of appearance of septal defect in CT and associated vascular abnormalities is critical for providing accurate information for the management and surgical approach [13]. 

Our study was aimed to assess vascular anomalies associated with SV-ASD using CT angiography. Our study included 72 patients over a period of 2.5 years. Age range of cases was from two months to 14 years. Mean age was 2.8 ± 2.7 years. A total number of male and female cases were 37 (51.3%) and 35 (48.6%), respectively; male to female gender distribution in SV-ASD is reported to be 1:1.3. Of 72 patients, 64 (88.8%) patients had superior sinus venosus atrial septal defect, while inferior sinus venosus atrial septal defect was found in eight (11.1%). Attenhofer Jost et al. reported superiorly located SV-ASD in 94.7% patients and inferior SV-ASD in 5.3% patients [19]. In seven (9.7%) patients, anomalous vein was draining into upper and middle part of SVC (Figure 1). Anomalous vein draining into the IVC was seen in nine (12.5%) patients. In one (1.3%) case, anomalous vein was seen opening into the right atrium. In 55 (76.3%) patients, anomalous pulmonary vein was seen draining at SVC and right atrial junction. In six (8.3%) patients, associated secundum atrial septal defect was identified. Persistent left superior vena cava was found in seven (9.7%) patients, while James et al. reported 8% of patients with persistent left superior vena cava associated with SV-ASD [20]. The significant clinical implication of persistent left SVC is that its diagnosis must be known before cannulation for open heart surgical repair or correction. In a study conducted by Lewis et al., out of 90 patients with PAPVR, 31 (34%) patients had associated SV-ASD [21]. In our patient population, the most common site of anomalous vein was seen draining into SVC and right atrial (RA) junction in 55 (76%) patients followed by nine (12.5%) in IVC and seven (9.7%) in upper and middle part of SVC, and in one (1.3%) patient, anomalous vein was seen draining directly into the RA. Left aortic arch was seen in 70 (97.2%) patients, while two (2.7%) patients had right aortic arch. The prevalence of right aortic arch is reported to be around 0.1% in general population [22]. Significance of this finding is that a right aortic arch is associated with more than twice risk of surgical complications [23].

This study was conducted at pediatric cardiology unit, and most of the patients were referred to other centers for surgical repair, because of lack of availability of pediatric cardiac surgery services at our hospital. Further studies are required in this regard including surgical findings to correlate with preoperation cardiac imaging findings.

## Conclusions

Sinus venosus ASD is a rare type of atrial septal defect which is also associated with both pulmonary and systemic vascular anomalies. Diagnosing these vascular anomalies is of paramount importance before any corrective intervention can be done. Recognizing the pattern of these anomalies should be known to every interventional cardiologist, radiologist, and cardiac surgeon. In our study, the commonest site of anomalous pulmonary venous return was at superior vena cava and right atrial junction. Persistent left superior vena cava was found in majority of patients. Superior sinus venosus ASD was the most common type of SV-ASD. Echocardiography alone is not a good tool to assess these extracardiac structures. Imaging modalities like CT angiography and MRI have refined our preoperative workup which is essential for the ultimate outcome of the corrective intervention. 
